# Tetra­kis(dimethyl­ammonium) *trans*-di­chloridobis[5,5′-(pyrazine-2,3-diyl)bis(1*H*-tetra­zol-1-ido-κ*N* ^1^)]copper(II)

**DOI:** 10.1107/S1600536812036896

**Published:** 2012-09-01

**Authors:** Ju-Hsiou Liao, Pei-Shan Shi

**Affiliations:** a Department of Chemistry and Biochemistry, National Chung Cheng University, 168 University Road, Min-Hsiung, Chia-Yi, Taiwan

## Abstract

The title compound, (C_2_H_8_N)_4_[Cu(C_6_H_2_N_10_)_2_Cl_2_], consists of an anionic complex which is composed of a Cu^II^ ion surrounded by four N atoms from two pyrazine-2,3-diylbis(1*H*-tetra­zol-1-ide) ligands, and two Cl^−^ atoms in a *trans*-Cl_2_N_4_ coordination geometry; the Cu^II^ atom lies on a site of symmetry 2/*m*. The Cu—Cl distance of 2.8719 (5) Å is long due to the Jahn–Teller distortion of the *d* ^9^ electron configuration of Cu^II^ ion. The tetra­zole and pyrazine rings make an N—C—C—N torsion angle of 38.25 (17)°. The charge of the anionic complex is balanced by four dimethyl­ammonium cations, which inter­act with the anionic complexes *via* N—H⋯N and N—H⋯Cl hydrogen bonds.

## Related literature

For the coordination compound of 2,3-di-1*H*-tetra­zol-5-yl­pyrazine, see: Li *et al.* (2008 ▶). For related structure, see Tao *et al.* (2010 ▶). 
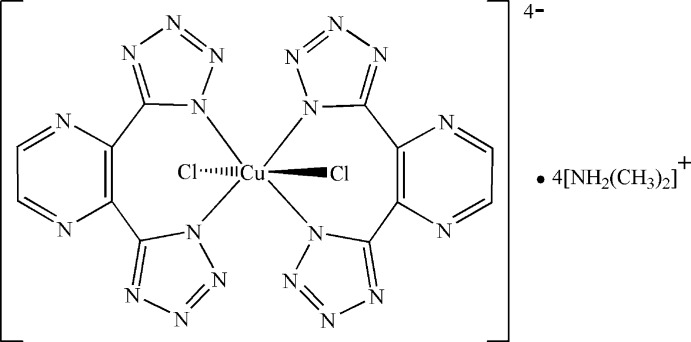


## Experimental

### 

#### Crystal data

(C_2_H_8_N)_4_[Cu(C_6_H_2_N_10_)_2_Cl_2_]
*M* *_r_* = 747.17
Orthorhombic, 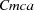

*a* = 20.613 (2) Å
*b* = 10.5671 (9) Å
*c* = 15.0687 (12) Å
*V* = 3282.3 (5) Å^3^
*Z* = 4
Mo *K*α radiation
μ = 0.89 mm^−1^
*T* = 293 K
0.06 × 0.06 × 0.05 mm

#### Data collection

Bruker SMART APEX diffractometer
Absorption correction: multi-scan (*SADABS*; Bruker, 1999 ▶) *T*_min_ = 0.947, *T*_max_ = 0.959
18389 measured reflections
2079 independent reflections
1888 reflections with *I* > 2σ(*I*)
*R*_int_ = 0.024

#### Refinement

*R*[*F* ^2^ > 2σ(*F* ^2^)] = 0.030
*wR*(*F* ^2^) = 0.085
*S* = 1.08
2079 reflections
120 parameters
H atoms treated by a mixture of independent and constrained refinement
Δρ_max_ = 0.74 e Å^−3^
Δρ_min_ = −0.26 e Å^−3^

### 

Data collection: *SMART* (Bruker, 1998 ▶); cell refinement: *SAINT* (Bruker, 1999 ▶); data reduction: *SAINT*; program(s) used to solve structure: *SHELXS97* (Sheldrick, 2008 ▶); program(s) used to refine structure: *SHELXL97* (Sheldrick, 2008 ▶); molecular graphics: *ORTEP-3* (Farrugia, 1997 ▶); software used to prepare material for publication: *SHELXTL* (Sheldrick, 2008 ▶) and *PLATON* (Spek, 2009 ▶).

## Supplementary Material

Crystal structure: contains datablock(s) I, global. DOI: 10.1107/S1600536812036896/pk2440sup1.cif

Supplementary material file. DOI: 10.1107/S1600536812036896/pk2440Isup2.cdx

Structure factors: contains datablock(s) I. DOI: 10.1107/S1600536812036896/pk2440Isup3.hkl

Additional supplementary materials:  crystallographic information; 3D view; checkCIF report

## Figures and Tables

**Table 1:** Selected bond lengths (Å)

Cu1—N1	2.0029 (10)
Cu1—Cl1	2.8719 (5)

**Table 2:** Hydrogen-bond geometry (Å, °)

*D*—H⋯*A*	*D*—H	H⋯*A*	*D*⋯*A*	*D*—H⋯*A*
N6—H6*B*⋯Cl1^i^	0.88 (2)	2.32 (2)	3.1731 (13)	162.7 (18)
N6—H6*A*⋯N4^ii^	0.93 (2)	1.91 (2)	2.8381 (17)	175 (2)

Symmetry codes: (i) 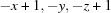; (ii) 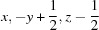.

**Fig. 1.**
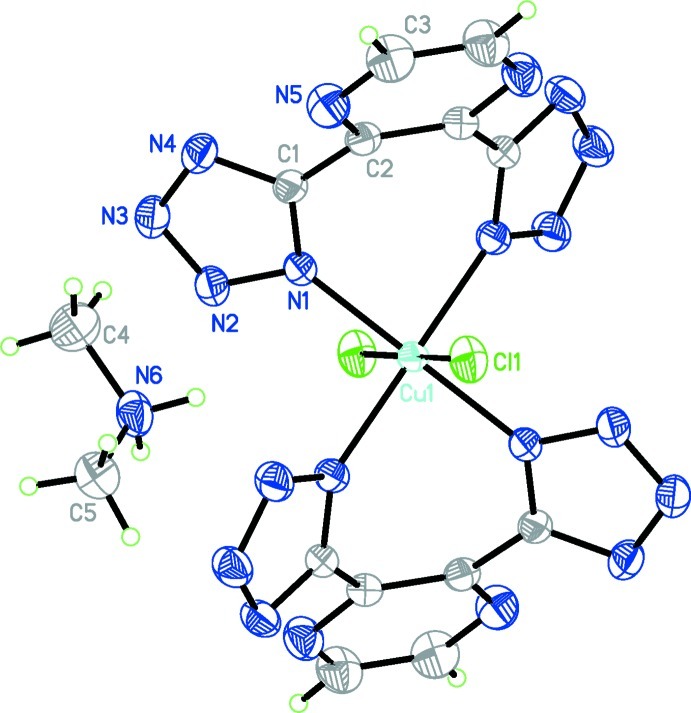
The structure of the title compound, with displacement ellipsoids drawn at the 50% probability level for non-H atoms. Unlabelled atoms are related to the reference atoms by the symmetry operations (1 - x, -y, 1 - z), (x, -y, 1 - z) and (1 - x, y, z).

**Fig. 2.**
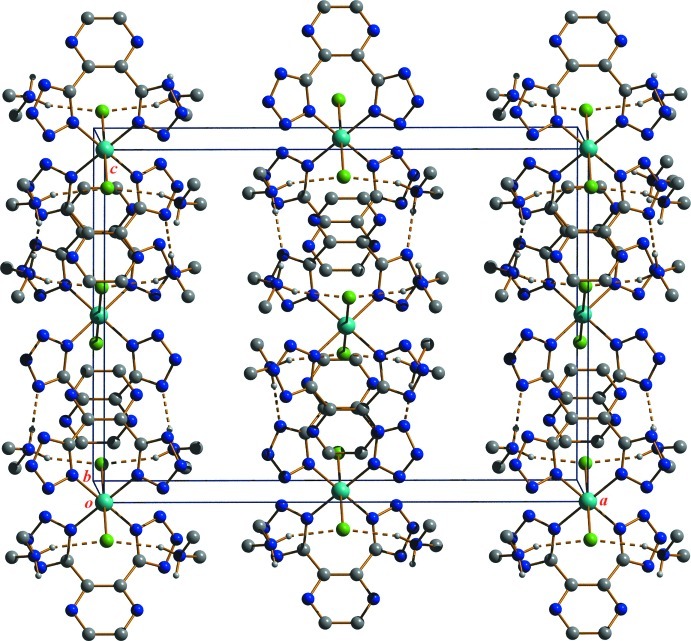
A packing diagram of the title compound. All H-atoms except for those involved in hydrogen bonds are omitted for clarity. Hydrogen-bonding interactions are drawn with dashed lines.

## supplementary crystallographic information

### Comment

Multifunctional tetrazolate ligands have recently been of great interest for the formation of metal-organic frameworks (MOFs). Thus far, the di-topic tetrazolate-based ligand, 2,3-di-1*H*-tetrazol-5-ylpyrazine (H_2_dtp) has only been found in a chiral, porous and thermally robust MOF, Zn(dtp) (Li, 2008). In our laboratory, the reaction of H_2_dtp and CuCl_2_ in dimethylformamide (DMF) under acidic conditions afforded the title compound (I).

In the title complex anion, the Cu^II^ ion is six-coordinated in a distorted octahedral environment, surrounded by two Cl^-^ anions and four N-atoms from two chelating (dtp)^2-^ anionic ligands, forming a *trans*-Cl_2_N_4_ coordination geometry (Fig. 1). The bonding mode is quite different from that observed in Zn(dtp). The asymmetric unit of the [CuCl_2_(dtp)_2_]^4-^ anion contains one quarter of the complex, with the Cu^II^ ion located at a site of 2/m symmetry, and the two Cl^-^ anions lie in a mirror plane. The Cu—Cl bond length, 2.8719 (5) Å, is unusually long due to Jahn-Teller distortion of the d^9^ electron configuration of Cu^II^ ion, while the Cu—N distance is normal at 2.0029 (10) Å. The tetrazolyl and pyrazinyl rings are not coplanar, with a torsion angle of 38.25 (17)°, in accord with the single-bond character of C1—C2 bond, 1.4678 (17) Å. In the aromatic CN_4_^-^ tetrazolate ring, the N2—N3 bond, 1.3071 (16) Å, has slightly more double bond character than those of N1—N2 and N3—N4 bonds, 1.3455 (15) Å and 1.3450 (17) Å.

Four equivalents of [(CH_3_)_2_NH_2_]^+^ cations are present to balance the charge, as shown in the packing diagram (Fig. 2). Slabs parallel to the *bc*-plane are formed by hydrogen bonding networks, which are constructed by the N—H bonds of [NH_2_(CH_3_)_2_]^+^ cations interacting with the Cl^-^ atoms and tetrazolate-N atoms of anionic complexes. Such slabs are stacked along the *a*-axis through van der Waals interactions among the methyl groups of the dimethylammonium cations.

### Experimental

4.3-mg (0.025 mmol) CuCl_2_^.^2H_2_O and 10.5-mg (0.05 mmol) H_2_dtp were dissolved in 1-ml dimethylformamide (DMF) respectively. The solutions were mixed in a reaction vial, adding 50-ml 3*M* HCl to adjust the pH value to ~1.5. The mixture was ultrasonicated to form a homogeneous yellowish green solution, and was kept at 120°C for three days. The product was washed with a small amount of DMF and acetone, and then dried in air. 18.2 mg of blue plate-like crystals were collected in 97.7% yield, based on Cu.

### Refinement

H atoms, except for H6A and H6B, were positioned geometrically and allowed to ride on their respective parent atoms with C—H = 0.96 Å [methyl, *U*iso(H) = 1.5*U*eq(C)] and C—H = 0.93 Å [aromatic, *U*iso(H) = 1.2*U*eq(C)]. H6A and H6B, which are involved in hydrogen bonds, were located in difference Fourier map and are refined freely. The highest peak (0.740 e Å^-3^) and the deepest hole (-0.257 e Å^-3^) in the difference Fourier map are located 0.79 Å and 1.19 Å from the atoms C2 and C3, respectively.

### Crystal data

**Table:**

(C_2_H_8_N)_4_[Cu(C_6_H_2_N_10_)_2_Cl_2_]	*F*(000) = 1548
*M**_r_* = 747.17	*D*_x_ = 1.512 Mg m^−^^3^
Orthorhombic, *C**m**c**a*	Mo *K*α radiation, λ = 0.71073 Å
Hall symbol: -C 2bc 2	Cell parameters from 999 reflections
*a* = 20.613 (2) Å	θ = 5–23.5°
*b* = 10.5671 (9) Å	µ = 0.89 mm^−^^1^
*c* = 15.0687 (12) Å	*T* = 293 K
*V* = 3282.3 (5) Å^3^	Hexagonal, blue
*Z* = 4	0.06 × 0.06 × 0.05 mm

### Data collection

**Table:**

Bruker SMART APEX diffractometer	2079 independent reflections
Radiation source: fine-focus sealed tube	1888 reflections with *I* > 2σ(*I*)
Graphite monochromator	*R*_int_ = 0.024
φ–ω scan	θ_max_ = 28.3°, θ_min_ = 2.0°
Absorption correction: multi-scan (*SADABS*; Bruker, 1999)	*h* = −27→26
*T*_min_ = 0.947, *T*_max_ = 0.959	*k* = −14→14
18389 measured reflections	*l* = −19→20

### Refinement

**Table:**

Refinement on *F*^2^	Primary atom site location: structure-invariant direct methods
Least-squares matrix: full	Secondary atom site location: difference Fourier map
*R*[*F*^2^ > 2σ(*F*^2^)] = 0.030	Hydrogen site location: inferred from neighbouring sites
*wR*(*F*^2^) = 0.085	H atoms treated by a mixture of independent and constrained refinement
*S* = 1.08	*w* = 1/[σ^2^(*F*_o_^2^) + (0.0517*P*)^2^ + 1.6198*P*] where *P* = (*F*_o_^2^ + 2*F*_c_^2^)/3
2079 reflections	(Δ/σ)_max_ = 0.001
120 parameters	Δρ_max_ = 0.74 e Å^−^^3^
0 restraints	Δρ_min_ = −0.26 e Å^−^^3^

### Special details

**Table:**

Geometry. All e.s.d.'s (except the e.s.d. in the dihedral angle between two l.s. planes) are estimated using the full covariance matrix. The cell e.s.d.'s are taken into account individually in the estimation of e.s.d.'s in distances, angles and torsion angles; correlations between e.s.d.'s in cell parameters are only used when they are defined by crystal symmetry. An approximate (isotropic) treatment of cell e.s.d.'s is used for estimating e.s.d.'s involving l.s. planes.
Refinement. Refinement of *F*^2^ against ALL reflections. The weighted *R*-factor *wR* and goodness of fit *S* are based on *F*^2^, conventional *R*-factors *R* are based on *F*, with *F* set to zero for negative *F*^2^. The threshold expression of *F*^2^ > σ(*F*^2^) is used only for calculating *R*-factors(gt) *etc*. and is not relevant to the choice of reflections for refinement. *R*-factors based on *F*^2^ are statistically about twice as large as those based on *F*, and *R*- factors based on ALL data will be even larger.

### Fractional atomic coordinates and isotropic or equivalent isotropic displacement parameters (Å^2^)

**Table:**

	*x*	*y*	*z*	*U*_iso_*/*U*_eq_	
Cu1	0.5000	0.0000	0.5000	0.02900 (12)	
Cl1	0.5000	−0.23652 (5)	0.59389 (3)	0.03605 (14)	
C1	0.42546 (6)	0.07284 (12)	0.66989 (8)	0.0248 (3)	
C2	0.46598 (6)	0.00989 (11)	0.73673 (8)	0.0253 (3)	
C3	0.46657 (8)	−0.09929 (14)	0.86700 (10)	0.0391 (3)	
H3	0.4447	−0.1387	0.9134	0.047*	
C4	0.32397 (9)	0.36802 (17)	0.42405 (12)	0.0474 (4)	
H4A	0.3170	0.3341	0.4824	0.071*	
H4B	0.2836	0.3985	0.4006	0.071*	
H4C	0.3545	0.4366	0.4273	0.071*	
C5	0.30596 (8)	0.15966 (16)	0.35528 (11)	0.0442 (4)	
H5A	0.3052	0.1116	0.4093	0.066*	
H5B	0.3210	0.1070	0.3076	0.066*	
H5C	0.2630	0.1894	0.3421	0.066*	
N1	0.43237 (5)	0.07041 (10)	0.58166 (7)	0.0266 (2)	
N2	0.38148 (6)	0.13511 (11)	0.54897 (7)	0.0323 (3)	
N3	0.34593 (6)	0.17510 (12)	0.61510 (8)	0.0342 (3)	
N4	0.37226 (6)	0.13670 (11)	0.69221 (8)	0.0312 (3)	
N5	0.43235 (6)	−0.04459 (12)	0.80258 (8)	0.0349 (3)	
N6	0.34981 (6)	0.26828 (14)	0.36567 (9)	0.0364 (3)	
H6A	0.3595 (11)	0.3006 (19)	0.3099 (15)	0.059 (6)*	
H6B	0.3887 (12)	0.2447 (19)	0.3834 (13)	0.053 (6)*	

### Atomic displacement parameters (Å^2^)

**Table:**

	*U*^11^	*U*^22^	*U*^33^	*U*^12^	*U*^13^	*U*^23^
Cu1	0.01762 (17)	0.0475 (2)	0.02182 (18)	0.000	0.000	−0.01293 (12)
Cl1	0.0268 (2)	0.0411 (3)	0.0402 (3)	0.000	0.000	0.0032 (2)
C1	0.0213 (6)	0.0300 (6)	0.0231 (6)	−0.0018 (4)	0.0021 (4)	−0.0041 (5)
C2	0.0256 (7)	0.0273 (6)	0.0231 (6)	−0.0004 (5)	0.0013 (5)	−0.0038 (4)
C3	0.0442 (8)	0.0416 (8)	0.0316 (7)	−0.0034 (7)	0.0052 (6)	0.0105 (6)
C4	0.0503 (10)	0.0485 (9)	0.0433 (9)	0.0003 (7)	0.0054 (8)	0.0000 (7)
C5	0.0405 (8)	0.0494 (9)	0.0428 (8)	0.0033 (7)	−0.0050 (7)	−0.0014 (7)
N1	0.0206 (5)	0.0374 (6)	0.0219 (5)	0.0022 (4)	−0.0014 (4)	−0.0042 (4)
N2	0.0238 (5)	0.0458 (7)	0.0274 (6)	0.0061 (5)	−0.0025 (4)	−0.0016 (5)
N3	0.0257 (6)	0.0451 (7)	0.0319 (6)	0.0078 (5)	0.0006 (4)	−0.0013 (5)
N4	0.0270 (6)	0.0386 (6)	0.0279 (5)	0.0055 (4)	0.0045 (4)	−0.0020 (5)
N5	0.0324 (6)	0.0413 (6)	0.0309 (6)	−0.0036 (5)	0.0041 (5)	0.0050 (5)
N6	0.0244 (6)	0.0565 (8)	0.0282 (6)	0.0034 (5)	0.0017 (5)	0.0067 (5)

### Geometric parameters (Å, º)

**Table:**

Cu1—N1^i^	2.0029 (10)	C4—H4A	0.9600
Cu1—N1^ii^	2.0029 (10)	C4—H4B	0.9600
Cu1—N1	2.0029 (10)	C4—H4C	0.9600
Cu1—N1^iii^	2.0029 (10)	C5—N6	1.469 (2)
Cu1—Cl1	2.8719 (5)	C5—H5A	0.9600
C1—N4	1.3309 (16)	C5—H5B	0.9600
C1—N1	1.3374 (15)	C5—H5C	0.9600
C1—C2	1.4678 (17)	N1—N2	1.3455 (15)
C2—N5	1.3405 (17)	N1—N2	1.3455 (15)
C2—C2^ii^	1.402 (3)	N2—N3	1.3071 (16)
C3—N5	1.3319 (19)	N3—N2	1.3071 (16)
C3—C3^ii^	1.378 (3)	N3—N4	1.3450 (17)
C3—H3	0.9300	N6—H6A	0.93 (2)
C4—N6	1.473 (2)	N6—H6B	0.88 (2)
			
N1^i^—Cu1—N1^ii^	180.0	H4B—C4—H4C	109.5
N1^i^—Cu1—N1	91.77 (6)	N6—C5—H5A	109.5
N1^ii^—Cu1—N1	88.23 (6)	N6—C5—H5B	109.5
N1^i^—Cu1—N1^iii^	88.23 (6)	H5A—C5—H5B	109.5
N1^ii^—Cu1—N1^iii^	91.77 (6)	N6—C5—H5C	109.5
N1—Cu1—N1^iii^	180.0	H5A—C5—H5C	109.5
N1^i^—Cu1—Cl1	88.82 (3)	H5B—C5—H5C	109.5
N1^ii^—Cu1—Cl1	91.18 (3)	C1—N1—N2	105.74 (10)
N1—Cu1—Cl1	91.18 (3)	C1—N1—N2	105.74 (10)
N1^iii^—Cu1—Cl1	88.82 (3)	C1—N1—Cu1	133.78 (9)
N4—C1—N1	110.40 (11)	N2—N1—Cu1	120.43 (8)
N4—C1—C2	121.69 (11)	N2—N1—Cu1	120.43 (8)
N1—C1—C2	127.80 (11)	N3—N2—N1	108.79 (10)
N5—C2—C2^ii^	121.15 (8)	N2—N3—N4	109.56 (11)
N5—C2—C1	114.09 (11)	N2—N3—N4	109.56 (11)
C2^ii^—C2—C1	124.68 (7)	C1—N4—N3	105.50 (10)
N5—C3—C3^ii^	121.98 (8)	C3—N5—C2	116.86 (13)
N5—C3—H3	119.0	C5—N6—C4	113.59 (13)
C3^ii^—C3—H3	119.0	C5—N6—H6A	108.9 (13)
N6—C4—H4A	109.5	C4—N6—H6A	110.8 (13)
N6—C4—H4B	109.5	C5—N6—H6B	111.8 (13)
H4A—C4—H4B	109.5	C4—N6—H6B	110.5 (13)
N6—C4—H4C	109.5	H6A—N6—H6B	100.5 (18)
H4A—C4—H4C	109.5		
			
N4—C1—C2—N5	38.25 (17)		

Symmetry codes: (i) *x*, −*y*, −*z*+1; (ii) −*x*+1, *y*, *z*; (iii) −*x*+1, −*y*, −*z*+1.

### Hydrogen-bond geometry (Å, º)

**Table:**

*D*—H···*A*	*D*—H	H···*A*	*D*···*A*	*D*—H···*A*
N6—H6*B*···Cl1^iii^	0.88 (2)	2.32 (2)	3.1731 (13)	162.7 (18)
N6—H6*A*···N4^iv^	0.93 (2)	1.91 (2)	2.8381 (17)	175 (2)

Symmetry codes: (iii) −*x*+1, −*y*, −*z*+1; (iv) *x*, −*y*+1/2, *z*−1/2.
